# Inherently Stealthy and Highly Tumor-Selective Gold Nanoraspberries for Photothermal Cancer Therapy

**DOI:** 10.1038/srep10311

**Published:** 2015-05-14

**Authors:** Naveen Gandra, Christopher Portz, Saide Z. Nergiz, Andrew Fales, Tuan Vo-Dinh, Srikanth Singamaneni

**Affiliations:** 1 Washington University in St. Louis, Department of Mechanical Engineering and Materials Science, 1 Brookings Dr., St. Louis, MO 63130; 2 Duke University, Departments of Biomedical Engineering and Chemistry and the Fitzpatrick Institute for Photonics, 101 Science Drive, Durham, NC 27708

## Abstract

Owing to their unique optical properties such as large absorption and scattering cross section and large enhancement of electromagnetic field at the surface, plasmonic nanostructures have received extensive attention as a highly promising class of materials for nano-oncology. Most of the existing plasmonic nanostructures require extensive post-synthesis treatments and biofunctionalization routines to mitigate their cytotoxicity and/or make them tumor-specific. Here, we report one-pot synthesis of a novel class of plasmonic nanostructures, namely, gold nanoraspberries (GRBs) with tunable size and localized surface plasmon resonance by using a naturally abundant polysaccharide, chitosan, which acts as a template and capping agent. Significantly, the GRBs, which do not require any further biofunctionalization, exhibit excellent selectivity to tumor cells, thus enabling locoregional therapy at the cellular level. We demonstrate the tumor-selectivity of GRBs by photothermal ablation of tumor cells selectively from their co-culture with normal cells. The simple, scalable and tumor-selective nature of GRBs makes them excellent candidates for translational plasmonics-based nanomedicine.

Nanomedicine holds great promise in revolutionizing the way we diagnose, image, and treat complex diseases such as cancer^1^,^2^,^3^,^4^,^5^,^6^,^7^,^8^,^9^,^10^,^11^,^12^,^13^,^14^,^15^. Progress over the last two decades has resulted in several nanoscale drug delivery systems such as Doxil, Daunoxome, Myocet, Albraxane, Genexol-PM that are approved for clinical applications^16^,^17^. For homing in on tumor sites, most of the above mentioned delivery systems rely on the enhanced permeation and retention (EPR) effect caused by the leaky vasculature and poor lymphatic drainage of the tumor^18^,^19^,^20^. The effectiveness of the EPR effect mainly depends on the colloidal stability and blood circulation time of nanostructures under physiological conditions, which necessitate the modification of these nanostructures with “stealth” coatings such as polyethylene glycol (PEG) brushes to delay their uptake by macrophages and prolong their blood circulation time^21^. Although the polymer coatings enhance the serum stability and blood circulation time, they also hinder the desired nanoparticle uptake by cancer cells^22^. Targeted delivery of nanostructures to tumor site often requires further modification of the nanostructures with disease recognition bioreceptors such as antibodies and aptamers. This modification requires additional steps such as production, purification, conjugation, and sterilization of nanotherapeutic platforms. These steps, especially at the nanoscale level, are very complex and expensive, which make it difficult to translate most of the nanotherapeutic platforms to clinical applications^23^. For example, trastuzumab for targeted therapy of HER2-positive metastatic breast cancer and HER2-positive gastric cancer, costs ~$70,000 for treatment of each patient^24^,^25^. These considerations highlight the need for easy-to-synthesize, biocompatible, highly stable and cancer specific nanotherapeutics.

Here, we report one-pot synthesis of gold nanoraspberries (GRBs) with localized surface plasmon resonance (LSPR) in the near infrared (NIR) therapeutic window (650–900 nm) using chitosan, a naturally abundant polysaccharide, as a biocompatible stabilizing agent, obviating the need for conventional toxic surfactants and multi-step ligand-replacement procedures (Fig. 1A). The GRBs synthesized using chitosan exhibited high (i) serum stability; (ii) biocompatibility; (iii) tunable optical properties; (iv) pH sensitivity; and (v) tumour selectivity, which are highly desirable for successful translation of plasmonic nanomedicine into routine medical practices.

## Results and Discussion

**Figure 1:**
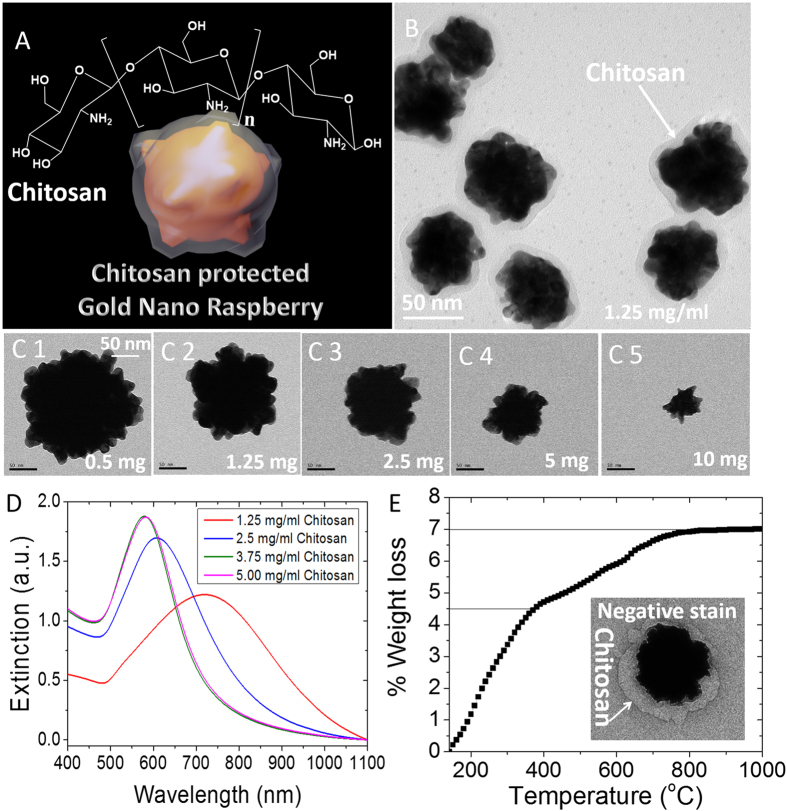
Chitosan stabilized gold nanostructures (**A**) Schematic representation of chitosan protected gold nanoraspberry and chemical structure of chitosan. (**B**) HRTEM image of GRBs (**C**1–**C**5) TEM images of GRBs synthesized with different concentration of chitosan (scale bars represent 50 nm). (**D**) Vis-NIR extinction spectra of GRBs synthesized with different concentrations of chitosan (**E**) Thermogravimetric analysis of GRBs to show percentage weight of chitosan and its transition temperature between 400 and 800 ºC. (Inset) TEM analysis with negative staining reveals 20–30 nm of polymer layer, which confirms the presence of chitosan on GRBs.

GRBs are synthesized using medium molecular weight (Mw~480,000 g/mol) chitosan (75–80% Degree of deacetylation (DD)) as a soft template and capping agent. To ensure complete solubility of chitosan in water, the pH of the aqueous solution was maintained below 6.0 (pKa of chitosan ~6.5). We noted that pH of the reaction significantly affects the rate, yield, and morphology of the GRBs. In a typical GRB synthesis, 50 μl of HAuCl_4_·4H_2_O (4.86 mM), 2.5 μl of AgNO_3_ (0.1 M), and 50 μl of ascorbic acid (0.1 M) were added to 10 ml of chitosan solution (1.25 mg/ml) at pH ~6. The color of the solution gradually turns to pale/dark blue within 10 minutes depending on the concentration of chitosan. The TEM images revealed the raspberry-like morphology of gold nanostructures obtained using this method (Fig. 1B). The GRBs were reasonably monodisperse with a diameter of 130 ± 13 nm. One of the important considerations in the design and synthesis of plasmonic nanostructures for *in vivo* biomedical applications is the ability to tune the LSPR of the nanostructures to NIR therapeutic window (650–900 nm), where the endogenous absorption coefficient of the tissue is nearly two orders magnitude lower compared to that in the visible part of EM spectrum. GRBs offer facile tunability of the size and optical properties, which makes them suitable for *in vivo* applications. The size of GRBs can be varied by altering the concentration of chitosan in the growth solution. Increasing the concentration of chitosan (from 0.5 to 5 mg/ml) led to a progressive decrease in the size of the nanostructures and a concomitant blue shift in the LSPR band of nanostructures (Fig. 1C,D). Interestingly, the characteristic raspberry morphology of these nanostructures is preserved across different sizes. These observations indicate the active role of chitosan in the formation of GRBs.

In the present study, we employed GRBs with ~130 nm diameter (synthesized using 1.25 mg/ml of chitosan) with LSPR peak at 780 nm. The strong optical absorption of GRBs in the therapeutic optical window makes them excellent candidates for photothermal therapy. In order to confirm the presence of chitosan layer and estimate the thickness of the same on GRBs, 2% uranyl acetate was used to negatively stain the TEM grids. TEM imaging revealed the presence of a ~20–30 nm polymer layer on GRBs (inset of Fig. 1E). The thickness of the chitosan layer on GRBs obtained from TEM analysis is consistent with the hydrodynamic diameter of GRBs measured using dynamic light scattering (Figure S4). To further estimate the amount of chitosan on GRBs, we performed thermogravimetric analysis (TGA) on GRBs powder. TGA revealed ~2.5% organic (chitosan) and 97.5% of inorganic (gold) content in the GRB sample, which is consistent with the TEM data (Fig. 1E).

**Figure 2:**
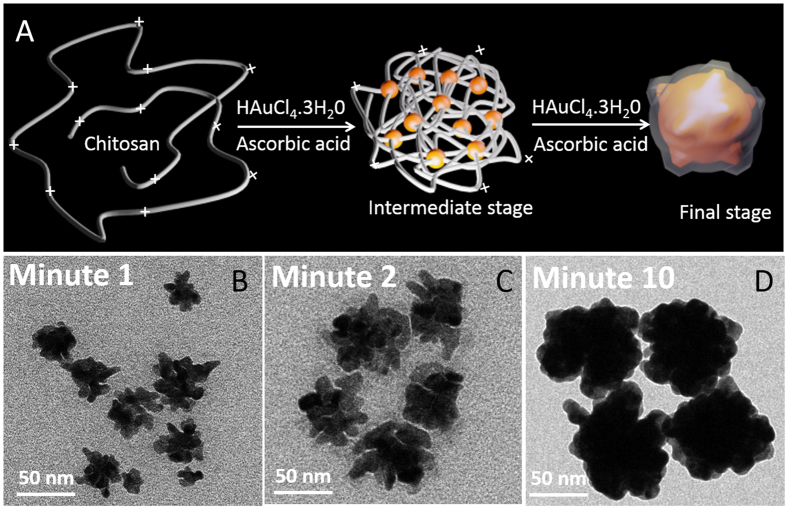
Proposed mechanism of GRBs formation (**A**) Schematic representation of GRBs formation from a highly open 3D polymer scaffold (**B**–**D**) TEM images of intermediate structures (t = 1, 2, and 10 minutes) at different stages of GRBs formation.

To understand the GRBs growth mechanism, TEM samples were prepared and analyzed at three different time points *i.e.,* 1, 2 and 10 min after the addition of ascorbic acid (reducing agent) into the growth solution (Fig. 2B–D). TEM images obtained after the first minute of growth revealed Au seeds that are not fully coalesced as evidenced by the tiny gaps within the branched nanostructures (Fig. 2B,C). Subsequently, these disconnected seeds continue to grow, leading to formation of GRBs as seen in TEM images at t = 2 and 10 min (Fig. 2C,D). Chitosan is a relatively stiff polymer with a large persistence length (10–25 nm), causing the polymer chain conformation to resemble a highly open 3D scaffold. The protonated amine groups of chitosan that are known to have high affinity to Au possibly act as nucleation sites, forming tiny Au seeds along the chain, which coalesce upon subsequent growth in order to form raspberry shaped Au nanostructures.

**Figure 3:**
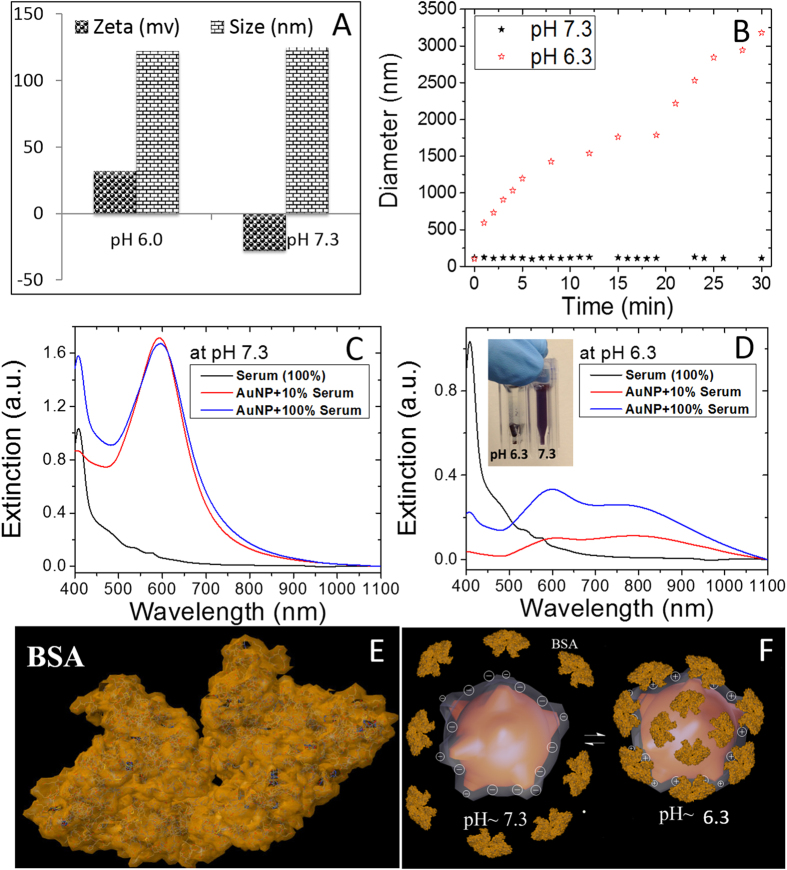
pH dependent serum stability of GRBs (**A**) Zeta potential and hydrodynamic size of GRBs at both physiological (~7.3) and tumorigenic (6.3) pH (**B**). Time dependent formation of protein corona on GRBs and subsequent aggregation of GRBs at pH 7.3 and 6.3. Vis-NIR extinction spectra of GRBs after incubating with 10% and 100% serum at (**C**) pH 7.3 and (**D**) pH 6.3. Inset of (**D**) shows that at pH 6.3, GRBs aggregate and sediment at the bottom of the cuvette. (**E**) X-ray crystal structure of BSA, an abundant protein in plasma (**F**) Schematic representation of protein corona formation at both physiological (~7.3) and tumorigenic (6.3) pH.

Any kind of nanoparticles intended for *in vivo* biomedical applications (*e.g.*, imaging and therapy) should possess high serum and plasma stability. In general, most of the naked and positively charged metal nanoparticles experience the formation of a protein corona once they are exposed to physiological fluids (Fig. 3B). The protein corona is known to trigger immune response, eventually leading to clearance of the nanoparticles from blood circulation^1^. Among other factors, the nature of protein corona on nanoparticles is governed by the size, shape, surface charge and surface chemistry of the nanoparticles^26^. Most of the nanoparticles developed so far require further processing to impart stealth character to nanoparticles. However, such strategies have resulted only in partial success, making their translation to preclinical and clinical settings difficult. Keeping this in mind, before proceeding to the *in vitro* experiments, we thoroughly investigate the serum stability of *as synthesized* GRBs without any surface modifications.

To understand the pH-dependent surface state of chitosan-coated GRBs, we measured their size and zeta-potential at both physiological (pH ~ 7.3) and tumorigenic (pH ~ 6.5) conditions. At physiological pH, GRBs exhibit a ζ-potential of −30 mV with an effective hydrodynamic diameter of 120 nm, whereas at pH ~6.3, the ζ-potential of the nanostructures is completely reversed to +30 mV with a hydrodynamic diameter of 120 nm (Fig. 3A). This pH dependent charge reversal behavior is similar to that exhibited by chitosan^27^, which further confirms the presence of chitosan on the GRBs. Now we turn our attention to the serum stability of GRBs dispersed in 10% and 100% FBS at pH ~7.3 and 6.3. As depicted by vis-NIR extinction spectra, even after 30 minutes of incubation at pH 7.3, the LSPR wavelength of GRBs did not exhibit any noticeable LSPR shift (Fig. 3C), which indicates their excellent stability and the fact that chitosan, under these pH conditions, effectively acts as a protein repellant (Fig. 3C). On the other hand, at pH 6.3, the extinction spectra of GRBs showed a dramatic change with the appearance of a broad extinction band at higher wavelength (~800 nm), which indicates aggregation of the nanoparticles in FBS as a result of protein corona around the nanoparticles (Fig. 3D). Even visual inspection of the nanoparticle solutions at these conditions confirms their stability and lack of thereof at pH ~7.3 and 6.3, respectively (Inset of Fig. 3D). To further understand protein corona formation and colloidal stability of GRBs, hydrodynamic diameter of these nanoparticles was monitored using dynamic light scattering (DLS) for the first 30 min after adding 10% FBS to nanoparticle solution. At pH 7.3, the hydrodynamic diameter of GRBs (~110 nm) remains virtually unchanged even 30 min after adding 10% FBS. On the other hand, at pH 6.0, hydrodynamic diameter of the GRBs monotonically increased up to 3 μm within 30 min, indicating the strong aggregation of the nanoparticles in solution (Fig. 3B). At low pH, the positively charged GRBs tend to interact with negatively charged serum proteins e.g., bovine serum albumin (BSA) (net charge of BSA in complete medium is −20 mV) as shown in Fig. 3F. Serum stability studies clearly indicate that the GRBs exhibit excellent stability at physiological pH (~7.3), which is important to escape the immune system and to maximize the blood circulation time. At the same time, poor colloidal stability of GRBs at tumorigenic pH (~6.3) causes them to preferentially accumulate at the tumor site^28^.

**Figure 4:**
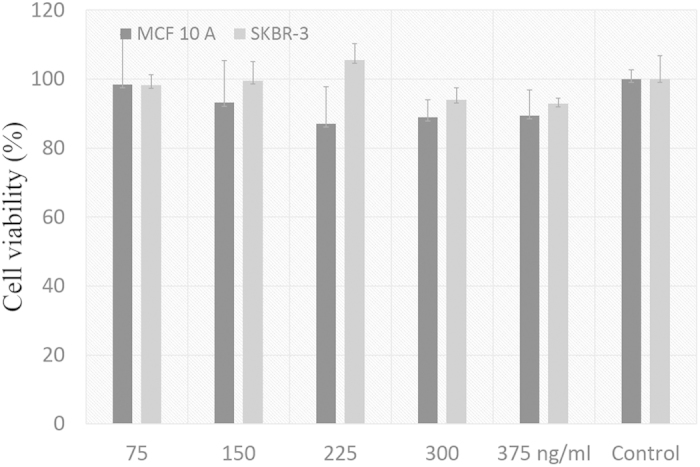
Cytotoxicity of GRBs No significant drop in cell viability was observed for both SKBR-3 and MCF-10A cells even at very high concentration of GRBs (375 ng/ml), which indicates the biocompatible nature of these nanoparticles.

MTT (3-(4,5-dimethylthiazol-2-yl)-2,5-diphenyltetrazolium bromide) assay was performed to evaluate the cytotoxicity of GRBs (75 to 375 ng/ml) in both MCF-10A (epithelial normal breast cells) and SKBR-3 (epithelial breast cancer cells) cells (Fig. 4). The concentration of GRBs was measured in nanoparticle tracking analysis (NTA) and verified with inductively coupled plasma mass spectrometry (ICP-MS). Both the cell lines show very high cell viability (>90%) over a wide concentration range (25 to 375 ng/ml) after 12, 24, 48 hrs of incubation with GRBs (Fig. 4, Figure S2 and S3). It is important to note that the trace amount of free chitosan in GRBs solution leads to higher cell viability of SKBR-3 cells. However, after complete removal of free chitosan in the solution, the cell viability drops to 90%, which is expected due to the oxidative stress caused by the metal nanoparticles (Fig. 4). In order to better understand the effect of free chitosan on the cell viability, MTT studies were conducted using different concentration of chitosan (0.075 to 250 μg/ml). Low chitosan concentration (up to 0.375 μg/ml) promoted the growth of SKBR-3 cells due to the overexpressed glycoproteins in cancer cells, but no major change was observed in MCF-10A cell viability. For higher concentrations of chitosan (>50 μg/ml), the viability of both SKBR-3 and MCF-10A cells significantly reduced (Figure S7). We believe that the higher concentration of chitosan blocks normal mechanisms of cell by interfering with surface receptors, which ultimately triggers the apoptosis in both SKBR-3 and MCF-10A cells.

**Figure 5:**
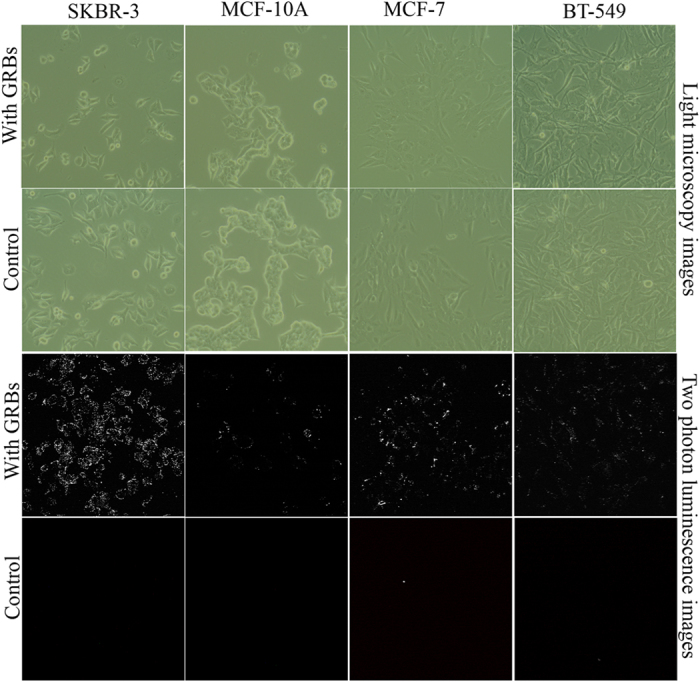
Mechanism of internalization studies Two-photon luminescence (TPL) of GRBs were measured using three different breast cancer cell lines SKBR-3, BT-549, MCF-7 and compared with MCF-10A, breast epithelial cells (**A**) Light micrscopy images of SKBR-3, MCF-10A, BT-549, and MCF-7 cell lines using the phase contrast mode after GRBs internalization in both passive and active targeting (**B**) TPL images of the same cell lines, which clearly indicated SKBR-3 cells exhibit maximum photoluminescence with active targeting whereas nonspecific adsorption was observed in control MCF-10A cells.

As mentioned above, selective delivery of nanotherapeutics to tumor sites is critical for successful administration of locoregional therapy. Polysaccharides are known to internalize into several cancer types that overexpress folate receptors (FR)^29^. However, to better understand the cancer selective internalization, we explored the internalization of GRBs in four different breast cancer cells (Fig. 5), MCF-10A (human mammary gland epithelial cells, FR- and ERBB2–), SKBR-3 (human breast carcinoma cells, FR+ and ERBB2+), MCF-7 (human breast carcinoma cells, FR− and ERBB2+)^30^, and BT-549 (human breast carcinoma cells, Triple negative but FR+ and ERBB2–). To check the internalization ability of GRBs in breast cancer, MCF-10A, SKBR-3, MCF-7, and BT-549 cells were incubated with GRBs for 4 hrs at 37° in a humidified atmosphere with 5% bone dry CO_2_. The short incubation time is selected to avoid passive targeting and to observe the effect of active targeting^31^. After 4 hrs of incubation with GRBs (100 μl of 0.1 nM), MCF-10A, SKBR-3, MCF-7, and BT-549 cells (4.0 × 10^5^ cells in a 6-well plate) were rinsed two times with PBS prior to two-photon luminescence (TPL) imaging. An Olympus FV1000 multiphoton system with tunable femtosecond Ti-sapphire laser (680–1080 nm) was used to record TPL spectra using a 40X water immersion objective. We performed the internalization studies in the presence of FBS, which is important for targeted delivery. The bright luminescence from the SKBR-3 cells indicates the presence of large number of GRBs (Fig. 5). On the other hand, in MCF-10A no substantial TPL was observed, which confirms the selective internalization of GRBs into SKBR-3 cells. The small number of bright spots from the MCF-10A cells is presumably due to the nonspecific adsorption of the GRBs on cell membranes. We also confirm the GRBs internalization studies using inductively coupled plasma mass spectroscopy (Figure S8) and TEM (Figure S9) analysis of sectioned cells. Interestingly, the GRB uptake levels between SKBR-3, MCF-7, and BT-549 [triple negative i.e., absence of epidermal growth factor receptor 2 (ERBB-2), estrogen receptors (ER), and progesterone receptors (PR)] show a significant difference. On the other hand, the difference in subcellular pH may also cause a difference in GRB uptake levels. Positively charged nanoparticles are known to have higher internalization compared to negatively charged nanoparticles^32^. Taken together, our internalization studies clearly indicate that chitosan-capped GRBs exhibit significantly higher internalization into SKBR-3 cells. However, the complete mechanistic aspects of cancer selectivity of GRBs require further investigations.

**Figure 6:**
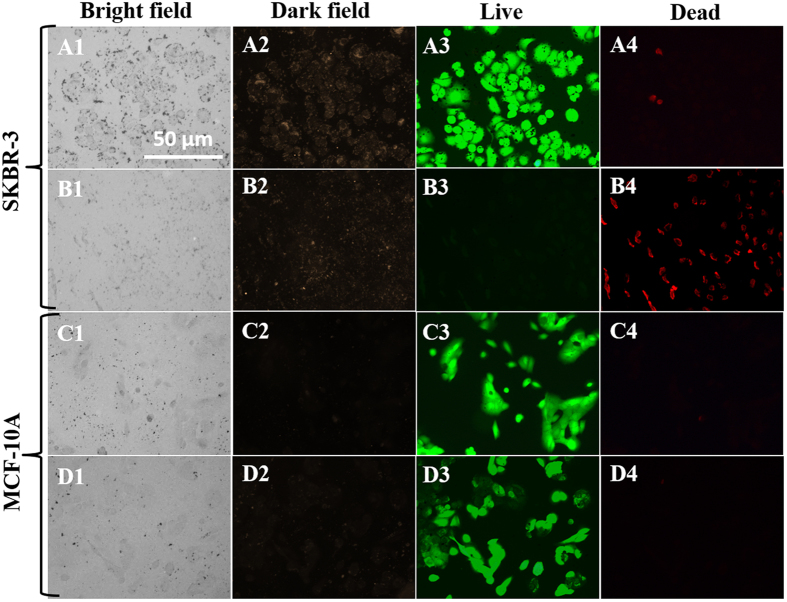
Photothermal Cancer therapy: Columns 1, 2, 3, and 4 are bright field, dark field, green fluorescence (live), and red fluorescence (dead) microscopy images, respectively. Rows **A**-**B** are SKBR-3 cells and Rows **C** - **D** are MCF-10A cells incubated with GRBs (10 ng/ml). Rows A and C correspond to cells not irradiated with laser and rows B and D correspond to those irradiated with laser. All unexposed cells shows only green fluorescence in column 3, which indicates that all the untreated cells are alive (i.e., GRBs alone do not result in any toxicity). In the case of exposed cells, only SKBR-3 cells are found to be dead as indicated by red fluorescence in B4 while the MCF-10A cells are unaffected by the treatment as indicated by the green fluorescence in D3.

**Figure 7:**
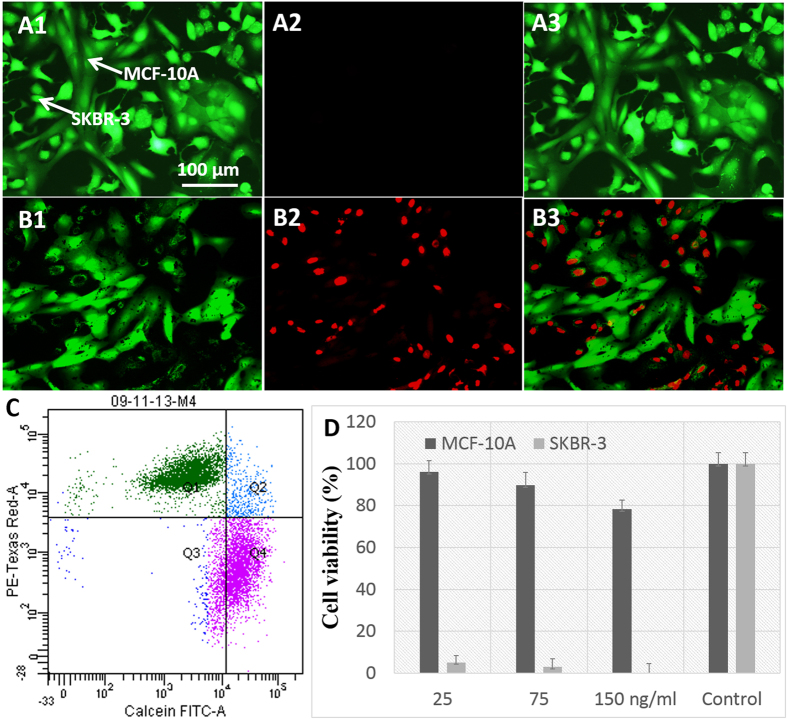
Selective photothermal therapy and quantification Fluorescence images of co-cultured cells with live/dead staining after laser exposure in the absence (**A**) and presence (**B**) of GRBs. (**C**) Flow cytometry of GRBs targeted co-cultured cells to quantify the number of live (pink) and dead (green) cells after photothermal treatment. (**D**) Viability of SKBR-3 and MCF-10A cells after photothermal therapy at different concentration of GRBs. After photothermal therapy, most of the SKBR-3 cells are dead even at very low concentration whereas 98% of MCF-10A cells are viable.

Once we confirmed the selective internalization of GRBs, we performed *in vitro* photothermal studies on MCF-10A, SKBR-3 and their co-cultures (Figs. 6,7). Here it is important to note that the cell death of both MCF-10A and SKBR-3 cells in co-culture were tested using both MTT and commercially available live/dead viability kit (green color for live and red for dead). Then photothermal studies with GRBs as contrast agents were performed on SKBR-3 and MCF-10A cell lines using the live/dead viability test kit as shown in Figs. 6,7. The cells in rows A-B and C-D in Fig. 6 correspond to SKBR-3 and MCF-10A, respectively. Both cell lines were incubated with 10 ng/ml of GRBs for 12 hrs prior to laser exposure (808 nm) (Fig. 6). Images in rows A and C correspond to cells that were not irradiated with laser. Images in rows B and D correspond to cells that were irradiated with laser at a power density of 320 mW/cm^2^ for 3 minutes. Fluorescence images were collected after exposing the cells to a live/dead staining solution for 30 min. The control cells, *i.e.,* cells that have been incubated with GRBs but not exposed to laser, exhibited bright green fluorescence, which corresponded to live cells and indicated that exposure to GRBs alone did not result in any significant cell death (Fig. 6A,C). Laser irradiation of cancer SKBR-3 cells that were incubated with GRBs resulted in significant cell death. On the other hand, laser irradiation of normal MCF-10A cells incubated with GRBs did not result in significant cell death as evidenced by the bright green fluorescence and absence of red fluorescence (Fig. 6D3). These observations are in good agreement with the GRBs internalization studies, which indicate the large uptake of GRBs by cancer SKBR-3 cells but absence of uptake by normal MCF-10A cells.

To further demonstrate the effectiveness of selective photothermal cancer therapy *in vitro*, selective cell killing experiments were conducted on co-cultures of SKBR-3 and MCF-10A cells that were incubated with GRBs (Fig. 7). As discussed above, due to the preferential uptake of GRBs into cancer cells, SKBR-3 cells were completely damaged after photothermal therapy as indicated by red fluorescence (Fig. 7B2). The green fluorescence in the same image indicated that MCF-10A cells remained alive (Fig. 7B3), which demonstrated that the photothermal therapy was highly selective to only breast cancer cells in this study. We also employed flow cytometry to count the numbers of live and dead cells after photothermal therapy in co-cultured cells. As depicted in Fig. 7C, 50 ± 5.55% of the cells were stained with red and 50 ± 3.82% of the cells stained with green, which further confirmed that half of the co-cultured cells were dead due to the selectivity of targeted photothermal therapy. This result is consistent with our live/dead fluorescence imaging of co-cultured cells after photothermal therapy. We also estimated cell viability after photothermal therapy using MTT studies (Fig. 7D). Even at very low concentration of GRBs, SKBR-3 cells were completely dead immediately after laser exposure whereas MCF-10A cells show ~95% viability. Taken together, photothermal studies performed on individual cell cultures and co-cultures demonstrated the effectiveness of GRBs for treating only cancer cells.

In conclusion, we demonstrate one-pot synthesis of inherently stealth and cancer-selective GRBs for photothermal breast cancer therapy using chitosan as an alternative to toxic surfactants. The GRBs shows unprecedented properties such as high serum stability, biocompatibility, tunable optical properties, pH sensitivity, and cancer selectivity, which are highly desirable for successful translation of plasmonics-based nanomedicine into routine medical practices in cancer care. While the mechanistic aspects of tumor-selective nature of GRBs need further investigation, our results indicate the excellent potential of these plasmonic nanostructures in administering locoregional therapy with minimal systemic toxicity.

## Materials

All materials were used as received without any further purification. Gold chloride (HAuCl_4_.4H_2_O), ascorbic acid, chitosan (medium molecular weight), 1-ethyl-3-(3-dimethylaminopropyl) carbodiimide (EDC), N-hydroxysuccinimide (NHS), fluorescein isothiocyanate (FITC), 3-(4,5-Dimethylthiazol-2-yl)-2,5-diphenyltetrazolium bromide (MTT) and pencillin-steptomycin were purchased from Sigma-Aldrich (St. Louis, MO, USA). Hydrochloric acid (HCl) was obtained from EMD (Gibbstown, NJ). Live/Dead Viability kit (Ethidium homodimer-1 and Calcein AM) and Trypsin-EDTA (0.25% 1X) were purchased from Life Technologies Corp. McCoy’s 5A medium, MEBM medium, MCF-10A cells, and SKBR3 cells were purchased from ATCC. MEGM bullet kit to mix with MEBM medium was purchased from Lonza (Kit Catalog No. CC-3150). The formvar/carbon coated copper TEM grids were acquired from Ted Pella (Redding, CA, USA). Nanopure water (>18.0 MΩ-cm) was used for all experiments.

## Methods

### Synthesis of chitosan protected gold nanoraspberries

The chitosan solution used in the synthesis of gold nanoraspberries was made by dissolving 50 mg of medium molecular weight chitosan in 3 mL of water at pH 1.4. Once the chitosan is completely dissolved after vigorous sonication and vortexing, an additional 7 mL of water was added to the concentrated chitosan solution, resulting in a final concentration of 5 mg/mL. The pH of the chitosan solution at this stage is close to 6.0. We then added 200 μL of the chitosan solution (5 mg/mL) to 800 μL of water and homogenized the solution by vortexing the solution. To this chitosan solution (1 mg/ml), 100 μL of gold chloride (4.86 mM) solution and 2.5 μL of silver nitrate (0.01 M) were added. The resultant solution was homogenized thoroughly to ensure the uniform solution. 50 μL of ascorbic acid (0.1 M) was added to the above reaction mixture under vigorous stirring (1200 rpm) for 30 sec. Then the solution was left undisturbed for overnight to form gold nanoraspberries.

### Cell culture

Human epithelial breast cells (MCF-10A) and breast cancer cells (SKBR3) were purchased from ATCC (Manassas, VA) and sub-cultured. MCF-10A cells were sub-cultured in base medium (MEBM) along with the additives obtained from Lonza/Clonetics Corporation (MEGM, Kit Catalog No. CC-3150). SKBR-3 cells were cultured in Mc.Coy’s 5A medium with 10% fetal bovine serum (FBS) and antibiotics (100 μg/ml penicillin and 100 μg/ml streptomycin) (Sigma, St. Louis, MO). BT-549 cells were cultured in RPMI-1640 complete medium (0.023 IU/ml insulin, fetal bovine serum to a final concentration of 10%). MCF-7 cells were cultured in Eagle’s minimum essential complete medium (0.01 IU/ml of recombinant insulin, fetal bovine serum to a final concentration of 10%). All cell lines were grown in water jacket incubator at 37ºC with 5% CO_2_-humidified atmosphere in 25 cm^2^ tissue culture flasks. Once the cells reached to 90% confluence, they were washed with phosphate buffered saline (PBS) and detached with 1 mL of 0.25% trypsin-EDTA solution (Sigma). Cells were dispersed in 10 ml complete medium with 10% FBS and centrifuged. Cells were counted in a disposable hemocytometer and plated at a density of 5 × 10^5^ and 4 × 10^4^ cells in flat bottom 24 well and 96 well plates (Corning life sciences), respectively. To co-culture, equal number (2 × 10^5^) of SKBR-3 and MCF-10A cells were plated in 24 well plates using MEBM as medium. MEBM did not cause any observable damage to SKBR-3 cells, which suggests MEBM can be used to culture both cell lines without significant cell damage.

### *In vitro* photothermal studies

Photothermal studies of MCF-10A, SKBR-3, and co-culture cells with and without gold nanoraspberries were conducted using 808 nm diode laser with a power density of 370 mW/cm^2^. At this power density, we did not observe any cell damage to either of the cells mentioned above, which indicates the laser power used is safe. To distinguish live and dead cells following the photothermal therapy, the cells were incubated with ethidium homobromide-1 and calcein AM dyes to produce green and red emission from live and dead cells, respectively.

### Characterization

TEM images were obtained using FEI sprint Lab6 with an accelerating voltage of 120 kV. HRTEM images were collected using FEI Tecnai G^2^ Twin with an accelerating voltage 200 kV. UV-vis-NIR extinction spectra were collected using a Shimadzu 1800 spectrophotometer. Hydrodynamic diameter and zeta potential of GRBs were measured using Dynamic Light Scattering (Malvern Zetasizer Nano S/ZS). Concentration of GRBs was measured by Nanoparticles Tracking and Ananlysis (NTA) using Nanosight instrument (NS500). Fourier Transform Infrared-Red spectra of GRBs and FITC-GRBs powder were measured using smart performer (attenuated total reflectance (ATR) accessory) in Nicolette Nexus 470. Thermogravimetric analysis of GRBs were performed by Q5000 IR thermogravimetric analyzer (TA instruments). Two photon luminescence images were collected using 40X water immersion long distance objective mounted to Olympus multiphoton imaging system with Tunable femto seconds Ti-Saph laser (680–1080 nm).

## Additional Information

**How to cite this article**: Gandra, N. *et al.* Inherently Stealthy and Highly Tumor-Selective Gold Nanoraspberries for Photothermal Cancer Therapy. *Sci. Rep.* **5**, 10311; doi: 10.1038/srep10311 (2015).

## Supplementary Material

Supplementary Information
