# Supplementary material for: Inherently Stealthy and Highly Tumor-Selective Gold Nanoraspberries for Photothermal Cancer Therapy
Source: Sci Rep. 2015 May 14;5:10311. doi: 10.1038/srep10311 (PMC4650759; doi:10.1038/srep10311)
Supplement: Supplementary Information [file srep10311-s1.doc]

Supporting information for

**Inherently Stealthy and Highly Tumor-Selective Gold Nanoraspberries for Photothermal Cancer Therapy**

Naveen Gandra, Christopher Portz, Saide Z. Nergiz, Andrew Fales, Tuan Vo-Dinh* and Srikanth Singamaneni*

*^1^Washington University in St. Louis, Department of Mechanical Engineering and Materials Science,1 Brookings Dr., St. Louis, MO 63130*

*^2^Duke University, Departments of Biomedical Engineering and Chemistry and the Fitzpatrick Institute for Photonics, 101 Science Drive, Durham, NC 27708*

*
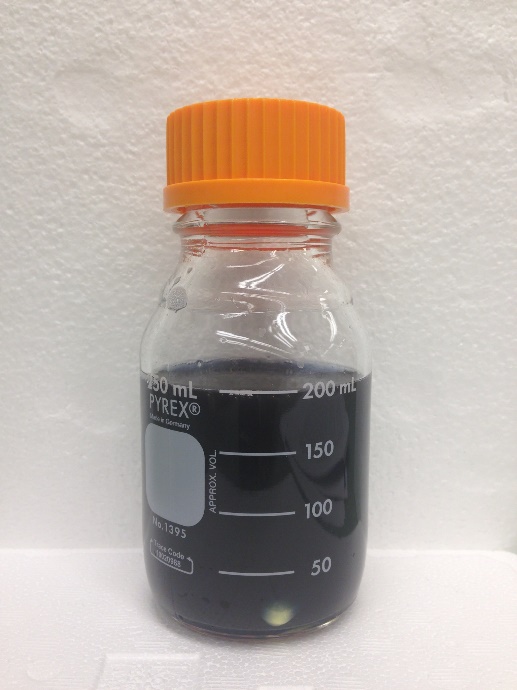
*

Figure S 1 One pot synthesis of 200 ml GRBs, which is important for tranlational nanomedicine.

*
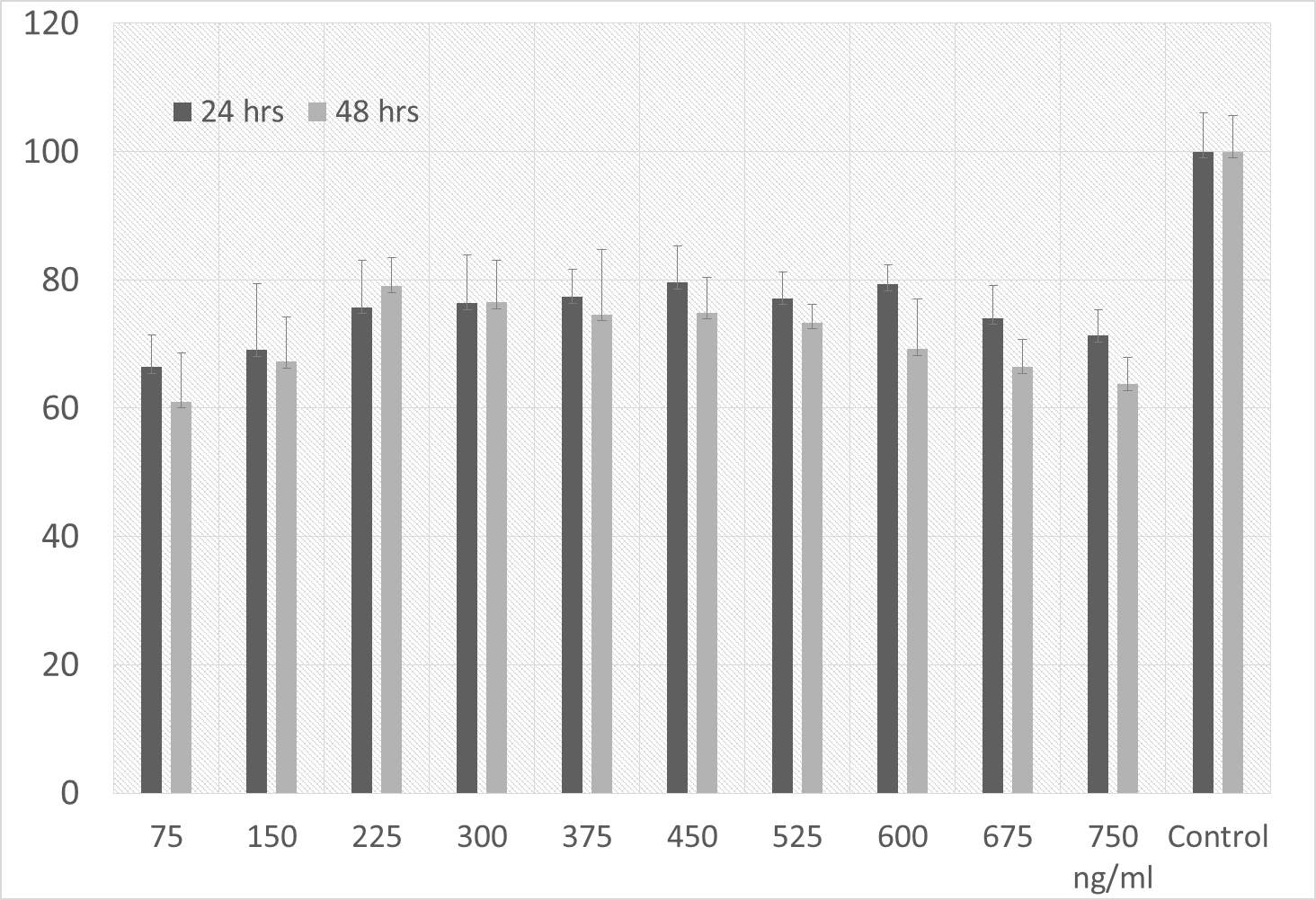
*

Figure S 2 MTT assay of SKBR-3 cell to determine the cell viability at different concentrations of GRBs.


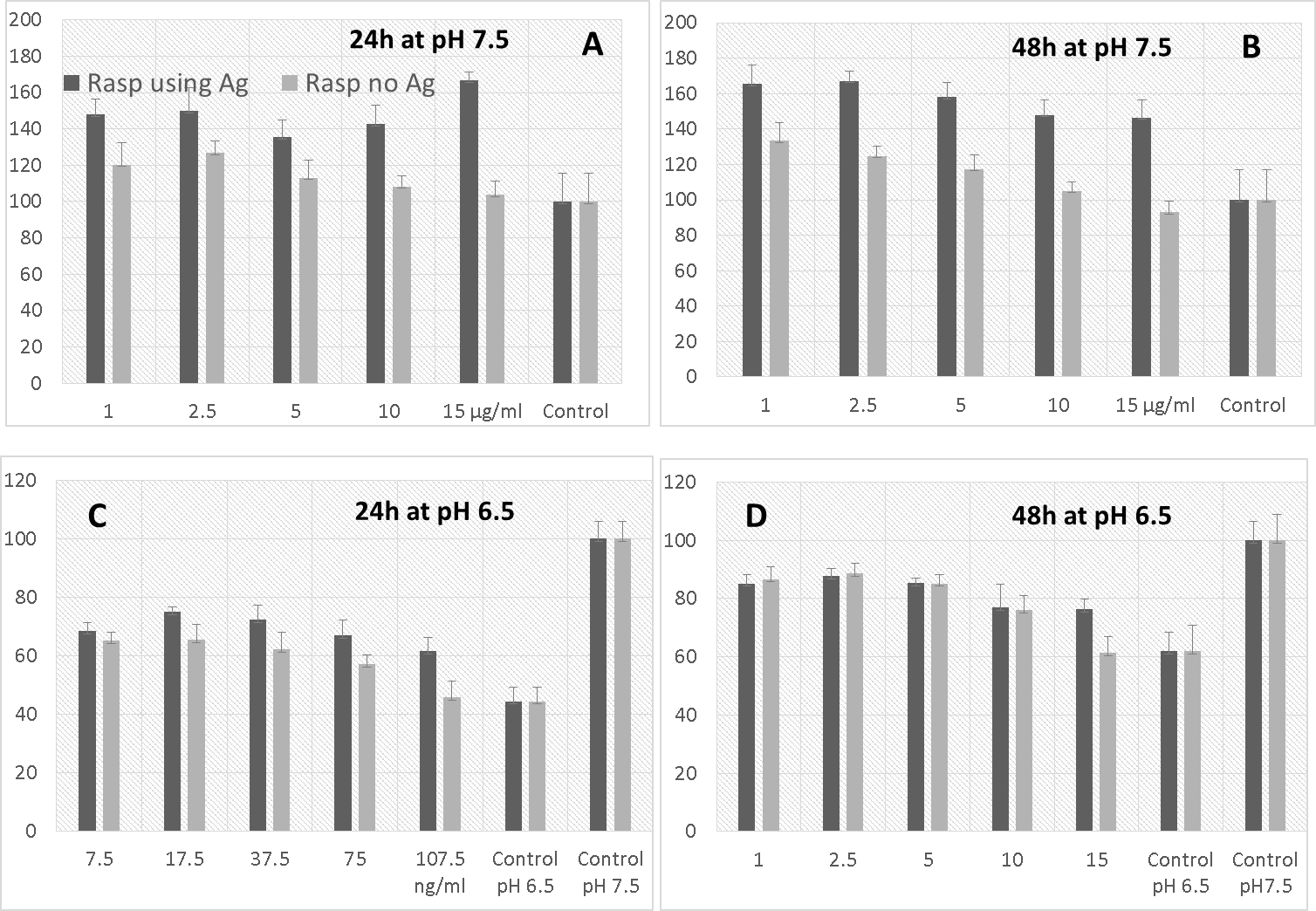


Figure S 3 Comparision of SKBR-3 cell viability in the presence of GRBs with and without Ag at both pH 7.5 and 6.5 after incubating for 24h and 48hrs.


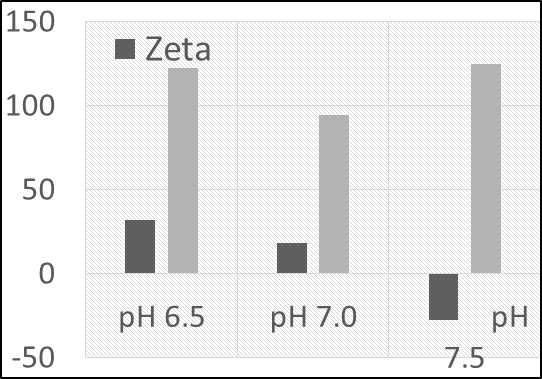


Figure S 4 Zetapotential of GRBs at pH 7.5(A) and pH 6.5 (B). Hydrodynamic size distribution of GRBs using Dynamic light scattering at pH 7.0 (red), pH 7.5 (blue), and pH 6.5 (black). Plot showing both zeta and size at pH 6.5, 7.0, and 7.5.

A

B

C

D

**EDC activated Fluorescein**

**pH~ 10**

Chitosan capped Fluorescein conjugated Gold Nano Raspberries

Chitosan capped

Gold Nano Raspberries

Figure S 5. Scheme showing the chemical conjugation of Fluorescein with Chitosan.

Figure S 6 Thermogravemetri analysis of GRBs between 200 and 1000 ^o^C to show the presence of percentage weight of chitosan. The organic content was burn between 400 and 800 ^o^C , which confirms the transition temperature of chitosan.

**
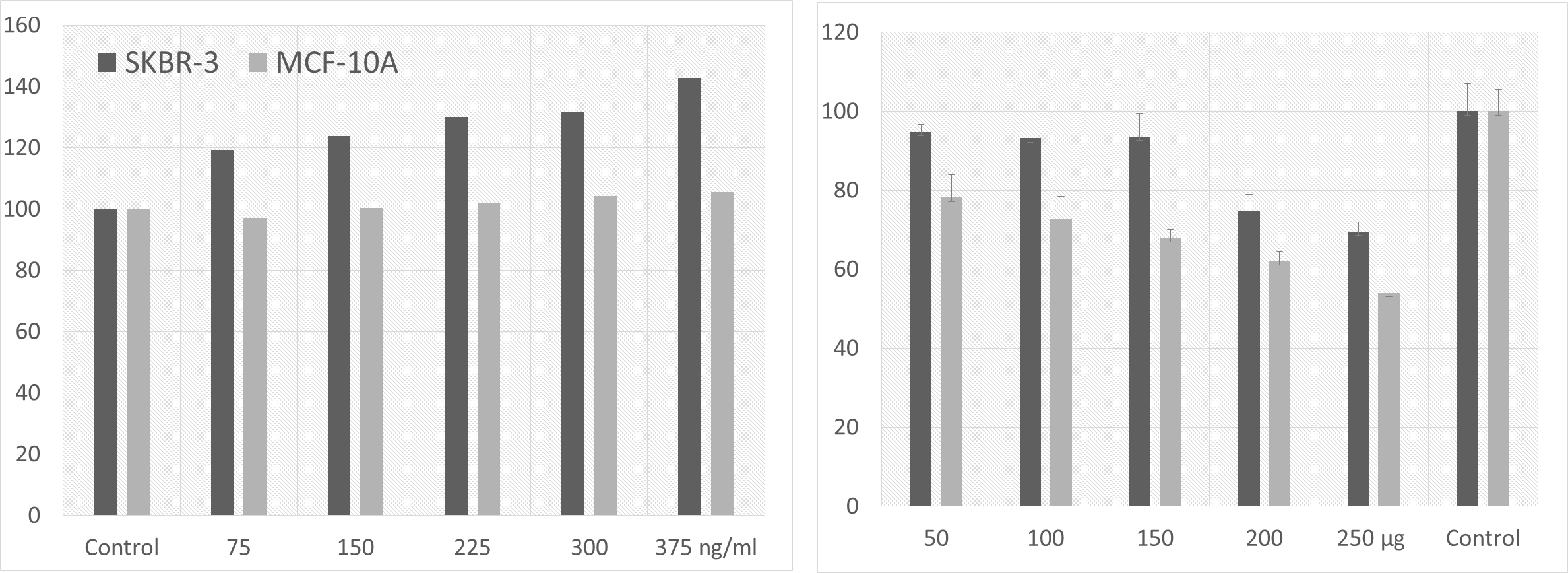
**

Figure S 7 Viability of SKBR-3 and MCF-10A cells in the precence of chitosan between 0.075 µg to 250 µg. Our results shows that between 0.075 and 37.5 µg, the viability of SKBR-3 cell higher than the control cells. But there is no significant difference in MCF-10A cells. Once the concentration levels increases to 50 µg, the cell viability of both the cell lines significantly droped.


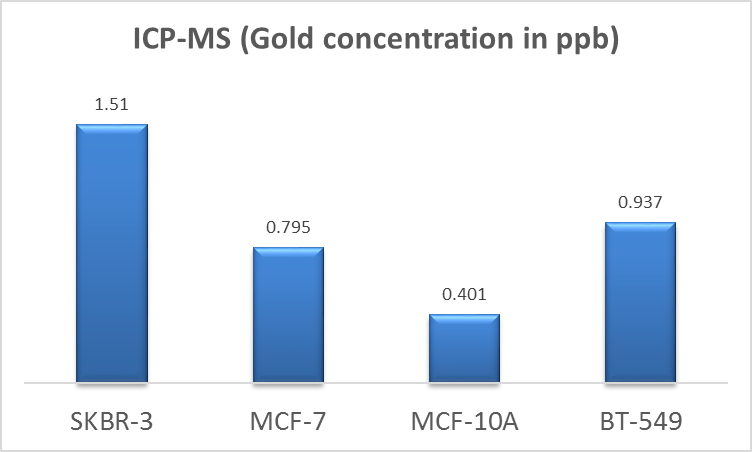


Figure S 8 Inductively coupled plasma mass spectroscopy (ICP-MS) was employed to measure the GRBs concentration in different cell lines.


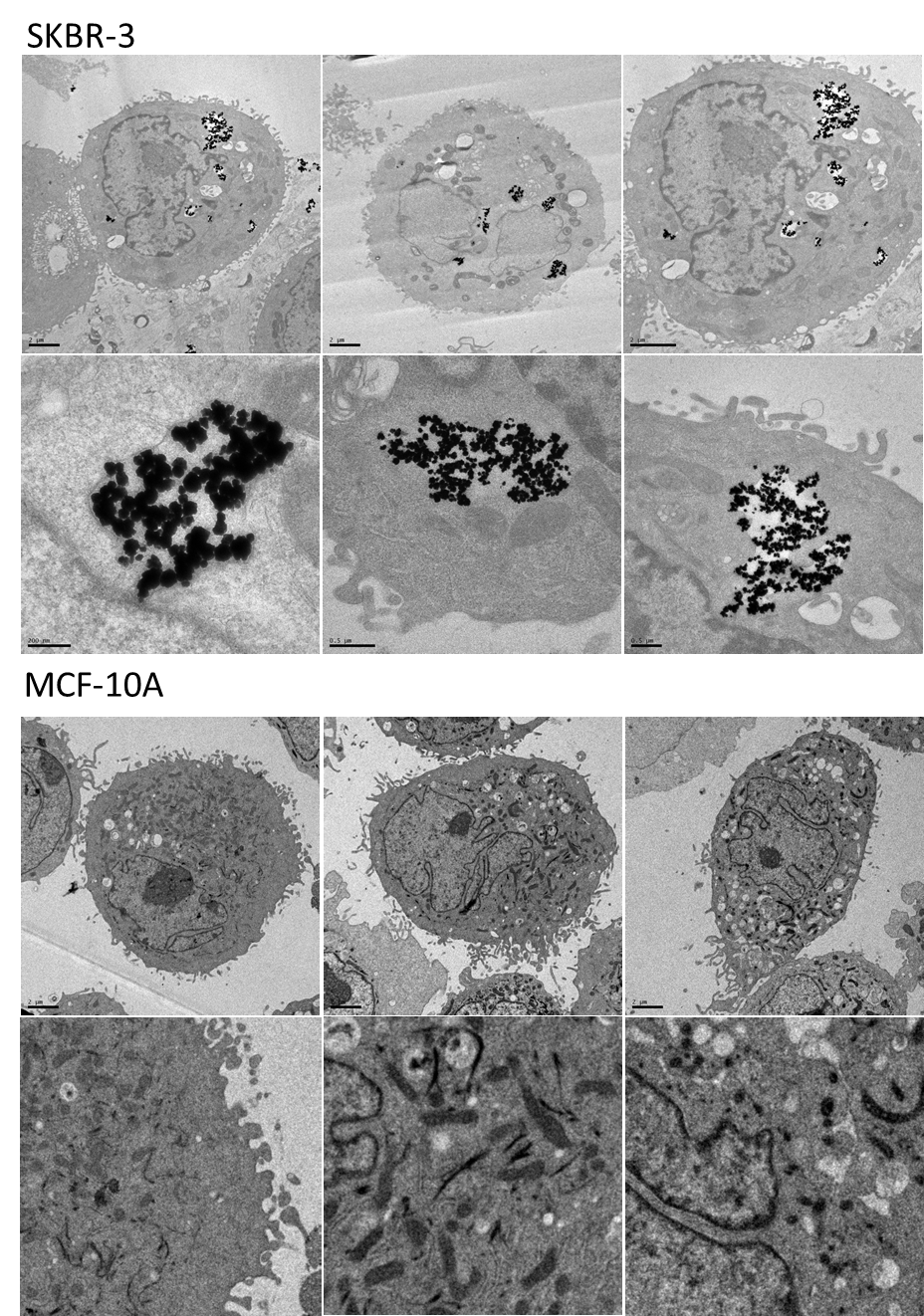


Figure S 9 TEM images of SKBR-3 and MCF-10A cells after incubation with GRBs. TEM sections clealry shows that SKBR-3 cells contains large number of GRBs, which indicates GRBs internalize in SKBR-3 cells. Although, some nonspecific adsorption absorved in TPL and ICP-MS experiments, GRBs are not internalized in MCF-10A cells.
